# Breaking barriers: using the behavior change wheel to develop a tailored intervention to overcome workplace inhibitors to breaking up sitting time

**DOI:** 10.1186/s12889-019-7468-8

**Published:** 2019-08-16

**Authors:** Samson O. Ojo, Daniel P. Bailey, Marsha L. Brierley, David J. Hewson, Angel M. Chater

**Affiliations:** 1 Institute for Health Research, University Square, University of Bedfordshire, Luton, Bedfordshire LU1 3JU UK; 2 Institute for Sport and Physical Activity Research, School of Sport Science and Physical Activity, University of Bedfordshire, Polhill Avenue, Bedford, Bedfordshire MK41 9EA UK

**Keywords:** Sedentary behaviour, Sitting time, Behaviour change wheel, Intervention, COM-B, TDF, BCTs

## Abstract

**Background:**

The workplace is a prominent domain for excessive sitting. The consequences of increased sitting time include adverse health outcomes such as cardiovascular disease and poor mental wellbeing. There is evidence that breaking up sitting could improve health, however, any such intervention in the workplace would need to be informed by a theoretical evidence-based framework. The aim of this study was to use the Behaviour Change Wheel (BCW) to develop a tailored intervention to break up and reduce workplace sitting in desk-based workers.

**Methods:**

The BCW guide was followed for this qualitative, pre-intervention development study. Semi-structured interviews were conducted with 25 office workers (26–59 years, mean age 40.9 [SD = 10.8] years; 68% female) who were purposively recruited from local council offices and a university in the East of England region. The interview questions were developed using the Theoretical Domains Framework (TDF). Transcripts were deductively analysed using the COM-B (Capability, Opportunity, Motivation – Behaviour) model of behaviour. The Behaviour Change Technique Taxonomy Version 1 (BCTv1) was thereafter used to identify possible strategies that could be used to facilitate change in sitting behaviour of office workers in a future intervention.

**Results:**

Qualitative analysis using COM-B identified that participants felt that they had the physical *Capability* to break up their sitting time, however, some lacked the psychological *Capability* in relation to the knowledge of both guidelines for sitting time and the consequences of excess sitting. Social and physical *Opportunity* was identified as important, such as a supportive organisational culture (social) and the need for environmental resources (physical). Motivation was highlighted as a core target for intervention, both reflective *Motivation*, such as beliefs about capability and intention and automatic in terms of overcoming habit through reinforcement. Seven intervention functions and three policy categories from the BCW were identified as relevant*.* Finally, 39 behaviour change techniques (BCTs) were identified as potential active components for an intervention to break up sitting time in the workplace.

**Conclusions:**

The TDF, COM-B model and BCW can be successfully applied through a systematic process to understand the drivers of behaviour of office workers to develop a co-created intervention that can be used to break up and decrease sitting in the workplace. Intervention designers should consider the identified BCW factors and BCTs when developing interventions to reduce and break up workplace sitting.

## Background

Due to modernisation of society and technological advancements, there is now heavy reliance on computers in the workplace resulting in occupations being less physically demanding and more sedentary [1, 2]. Sedentary behaviour is any waking activity, such as sitting, reclining or lying which expends less than 1.5 metabolic equivalents [3]. From an operational standpoint, prolonged sitting at a desk is the type of sedentary behaviour typically observed in the office workplace. Seventy-three percent of the UK population aged 16–64 are currently in employment [4, 5] with a large number of these workers in office-related jobs [6]. Studies have identified that the workplace contributes to the majority of excessive daily sitting time in office-based employees [7, 8]. Self-reported occupational sitting time has been estimated at 6 h 30 min (IQR = 6 h 20–6 h 45 min) on a work day [9], which is in accordance with objective measurements of workplace sitting suggesting 71% [10] to 82% of the workday is spent seated [11]. Due to growing epidemiological evidence linking excessive sitting time to adverse cardiometabolic outcomes, such as cardiovascular disease, obesity, type 2 diabetes [6, 12–17] and poor mental wellbeing [13, 18–20], the workplace has become an important public health concern.

Two observational studies have shown that daily participation in moderate-to-vigorous physical activity (MVPA) for 60–75 min a day may eliminate the increased risk of premature mortality associated with high amounts of sitting [21, 22]. However, the majority of the population do not engage in such high levels of MVPA [23, 24]. For those who are unable to achieve these high levels of MVPA, and in order to mitigate the remaining cardiometabolic health risks, the workplace could be a potential intervention environment to break up and reduce excessive sitting [25–27]. To develop effective interventions to reduce and break up sitting, it is pertinent to understand what works and why [28].

## Theoretical framework underpinning the intervention design

Interventions targeted at changing behaviour need to be informed by theoretical, evidence-based frameworks. The Medical Research Council [29] has outlined recommendations that should be used when developing and evaluating complex interventions. These guidelines state that interventions should start with a theory phase before progressing to modelling and then an experimental phase [29–31]. Whilst this current work focuses on modelling, the theory phase involves the collection of evidence and analyses via theoretical frameworks through which an intervention can be developed and modelled. The modelling stage involves hypothesising what should be targeted (determinants of behaviour) and how this can be achieved (via behaviour change techniques) [32]. A wide range of theoretical models of behaviour have been developed including the Theory of Planned Behaviour [33] and the Health Belief Model [34]. One common limitation of these theories is that they only help to understand or predict behaviours [35] and do not help to understand behaviour change [36] or develop interventions.

**Fig. 1:**
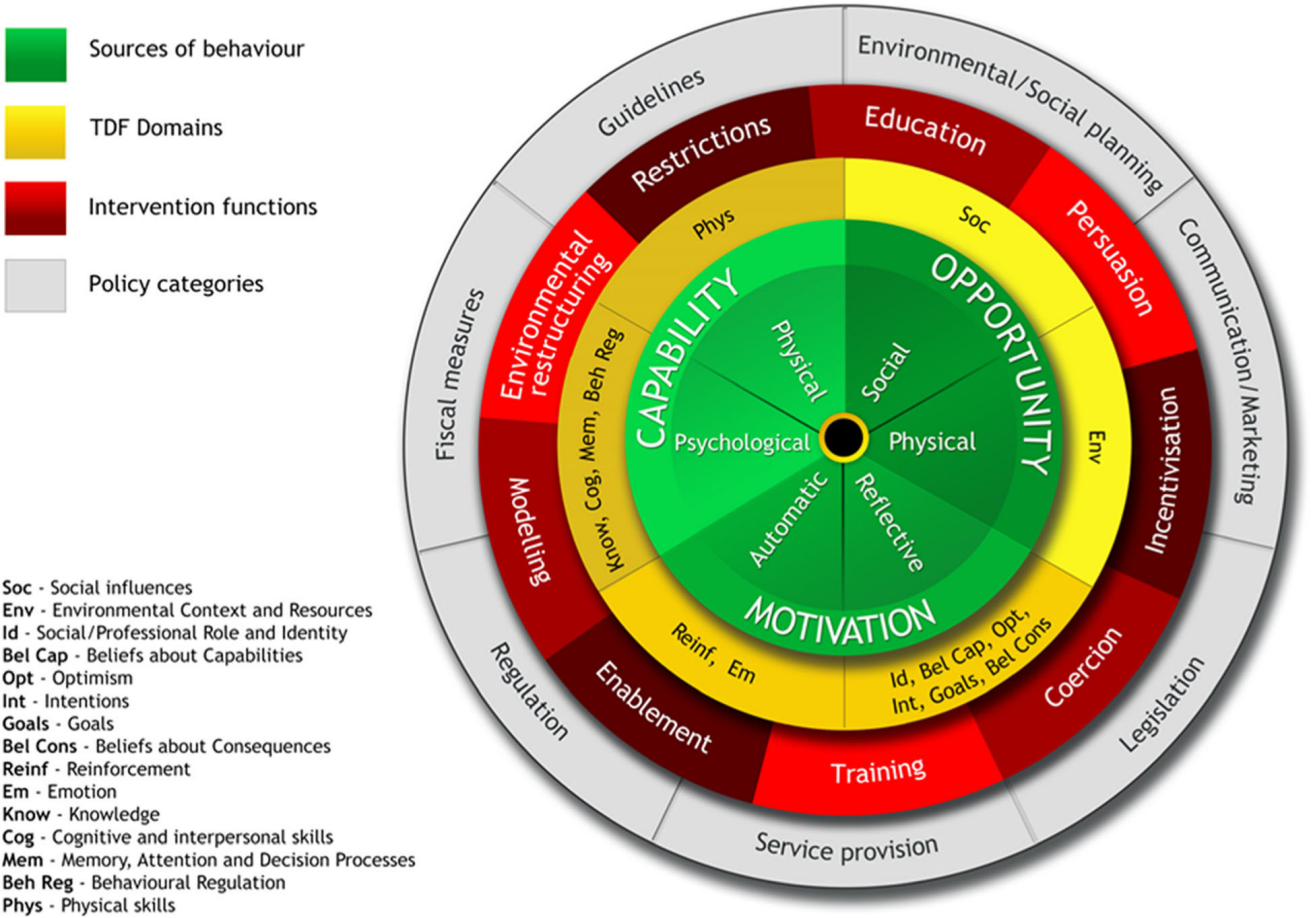
The Behaviour Change Wheel (reproduced with written permission from Michie, Atkins, et al. [37]). Protected by copyright

In order to help researchers transition from the behavioural diagnosis of a problem to the design of an intervention, the Behaviour Change Wheel (BCW) was developed [37, 38] from 19 behaviour change frameworks. At the hub of the BCW is the COM-B model (Fig. 1), addressing Capability, Opportunity, and Motivation sources of Behaviour. The BCW recognises that behaviour change occurs as a result of an interacting system with intervention functions and policy categories as the second and outer layer of the wheel [38]. The Theoretical Domains Framework (TDF) [39] has since been added to the BCW [40, 41] in order to help unpack COM-B further and allow deeper exploration of the barriers to and facilitators of change. The TDF includes constructs drawn from 33 behaviour change/psychological theories, to increase the understanding of behaviour to ensure the processes for change are targeted effectively [42]. The TDF has 14 domains (‘Knowledge’, ‘Skills’, ‘Social/Professional Role and Identity’, ‘Beliefs about Capabilities’, ‘Optimism’, ‘Beliefs about Consequences’, ‘Reinforcement’, ‘Intentions’, ‘Goals’, ‘Memory, Attention and Decision Processes’, ‘Environmental Context and Resources’, ‘Social Influences’, ‘Emotions’ and ‘Behavioural Regulation’ [39]). It has been used to identify factors that predict adherence to guidelines and for structuring both interview questions [43, 44] and how data analysis are performed [45, 46]. Once detail from the BCW and TDF have been obtained, optimal behaviour change techniques (BCTs) can be identified [47].

While it is important to identify how a behaviour maps to COM-B, the intervention functions (that form the third layer of the BCW) selected as a result must also make practical considerations. One method that has been developed to assist researchers to narrow down feasible intervention functions is to consider Affordability, Practicability, Effectiveness and Cost-effectiveness, Acceptability, Side effects/safety and Equity through the APEASE criteria [37]. Use of these criteria allow researchers to look beyond the BCW and explore feasibility issues before trialling an intervention. Using the BCW to design interventions is becoming more common, and it has been successfully used to understand behaviour change in different contexts, such as sexual counselling [48], medication management [49, 50], auditory rehabilitation [51], and physical activity [52]. Development of interventions using the full BCW to reduce workplace sitting however, is limited, with only the Stand More AT Work (SMArT) study found to target hospital office workers [53]. Ten behaviour change techniques that could be used to target individual, environmental and organisation level barriers to reducing sitting were identified in the SMArT study [53]. This intervention decreased workplace sitting time by 50.6 min at 3 months and by 64.4 min at 6 months and with evidence of sustainable long-term positive effects on job performance [54]. Therefore, further studies using this BCW framework are needed. The aim of this work is to develop, through qualitative interviews, a tailored intervention package using the BCW that could be used in future interventions to reduce and break up sitting time in desk-based employees.

## Method

**Fig. 2:**
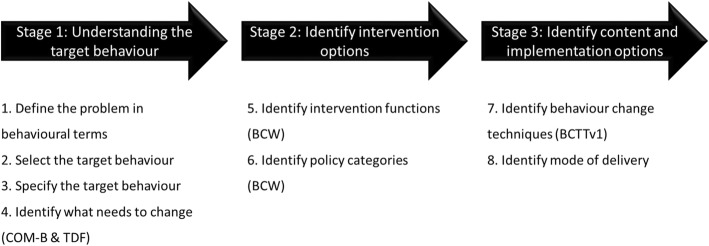
Stages involved in the development of an intervention using the BCW [37]

Ethical approval was obtained from the University of Bedfordshire Institute for Health Research Ethics Committee (approval number IHREC610). The processes of intervention development have been broadly categorised into three stages over eight steps as recommended for the BCW [37] and illustrated in Fig. 2. This study briefly describes steps one through three for contextual purposes but focuses on steps four through eight for intervention development.

## Step 1: define the problem in behavioural terms

The first step involves defining the problem of interest that requires intervention in behavioural terms. This means identifying the problem, and specifying the behaviour and target population [37]. Previous evidence [10, 11, 55–58] suggests that increased sedentary time is a behavioural problem significantly associated with cardiometabolic risk and poor mental wellbeing [12, 16, 59]. With office workers engaging in sitting for approximately two-thirds of their total working time and their sitting bouts often lasting at least 30 min [6, 11, 60, 61], there are possibilities that the workplace may be a major contributor to increased cardiometabolic disease risk.

## Step 2: select the target behaviour

This step explains that long lists of all other behaviours that may influence the target behavioural problem need to be generated. This can then be systematically reduced by considering the possible impact of each of these behaviours. For this research, behaviours such as physical activity, sedentary behaviour and sitting time were considered.

## Step 3: specify the target behaviour

Step three specifies the target behaviour by outlining the new behaviour in greater detail. Specifications should include: who needs to perform the behaviour, what do the persons need to do differently, when, where, how, and with whom will they do it [37]. In this research, the target behaviour is to break up and reduce sitting time at work which may follow guidelines in a recent expert statement [62], which states that office workers should initially reduce daily occupational sitting time by engaging in 2 h of standing or walking during working hours and gradually increasing this to 4 h per working day.

## Step 4: identify what needs to change

The recommended method to understand what needs to change is interviews or focus group discussions [63], as this would ensure future interventions are participant-centred and co-created [64]. This research aims to inform Step 4 by using semi-structured interviews to explore sitting behaviour in office workers drawing from both the COM-B and TDF. To achieve this, 25 office-based workers (26–59 years) with self-reported daily occupational sitting time of at least 5.5 h were purposively recruited from local council offices and a university in the East of England region and interviewed by one researcher (SO) in their respective offices. Questions asked in the interview were developed using the TDF [39] with each lasting between 30 to 60 min. Amendments were deemed not necessary following pilot interviews with three of the participants. Two researchers (SO & AC) independently coded the transcripts and maintained anonymity throughout by using pseudonyms [65]. The COM-B model and TDF were employed as a combined deductive framework for the analysis covering all the relevant determinants of behaviour [66, 67]. Comparisons of codes were made, and discrepancies resolved by discussion to produce ‘behavioural diagnosis’ (a selection of barriers and facilitators) for breaking up and reducing prolonged sitting in the workplace. The interview data was managed using NVivo qualitative data analysis software (Version 10, QSR International, Melbourne, Australia) while SPSS (Version 23, IBM, Chicago, IL, USA) was used for descriptive data analysis of participant characteristics.

## Step 5 and 6: identify intervention functions and policy categories

This study also aimed to identify relevant intervention functions and policy categories to be used following the COM-B and TDF analyses and how each of the intervention functions could be supported at an organisational level [37]. The BCW guide recommends that intervention functions and policy categories should be assessed through the use of the APEASE criteria [37]. However, as this screening process is largely contingent on resource availability, which might be different for intervention developers, the onus to use APEASE criteria would lie on individual intervention developers. In this present study, relevance of APEASE criteria is highlighted but not applied.

## Step 7 and 8: identify behaviour change techniques and mode of delivery

The research finally aimed to identify the most appropriate BCTs that could result in the desired breaking up and reduction of workplace sitting. BCTs mentioned within the qualitative interviews were individually identified and selected for the development of a future intervention by two members of the team (SO and MB). These were then discussed with the rest of the research team led by AC for consensus. Then, the most appropriate mode of delivery of each technique was deliberated upon and selected by the authors. Examples of modes of delivery include face-to-face or distance delivery at the individual or group level via phone (voice or text), print or digital media, broadcast media, outdoor media, or individually accessed computer programmes [37].

## Results

Steps 1–3 have been described in the methods above. This research has generated new qualitative data from Steps 4–8 as described below.

### Demographics of interview participants

The average age of the individuals who participated in the interviews was 40.9 ± 10.8 years, of which 68% were women, 56% White British, 68% university employees, and with an overall average body mass index of 25.7 ± 3.5 kg/m^2^.

## Step 4: identifying what needs to change

Responses from the interviews have been categorised into capability, opportunity and motivation in line with the COM-B model and includes links to the TDF domains within the text.

### Capability

The majority of participants said they are **physically capable** of breaking up their sitting time, although some highlighted that walking and standing trigger back problems.

*“I’m quite capable and confident of breaking up my sitting time. I do that quite a lot … .” (Participant 15, female, in their 20’s)*.

*“I’m sat down to help me improve my back muscles because standing or walking for too long can be detrimental for me” (Participant 24, female, in their 40’s).*

With respect to **psychological capability**, all participants stated that it was important to understand how much sitting is acceptable or excessive, as well as the consequences of prolonged sitting and any benefits of breaking up sitting time. This highlighted *knowledge* and *skills* as important TDF domains that should be targeted in an intervention:

*“If I'm really honest, I don't really know any current advice other than it's not good to sit down for too long … I think it would help if this is properly communicated” (Participant 10, female, in their 40’s).*

Most of the participants reported being engrossed in their work to meet tight deadlines, and this usually leads to them forgetting to take breaks from sitting. However, some participants believed that having a device or an app to remind them would help them to be more conscious, reflecting the TDF domain *memory, attention and decision processes.* In contrast, some participants said their sitting behaviour would change if they were able to monitor it by themselves, underlining the need for interventions to target the *behavioural regulation* TDF domain.

*“It’s just the amount of work, purely the amount of work that’s there. Also, not remembering to, because sometimes you become engrossed in a project, or in a piece of work … , your head is just focused on that piece of work … . It's a case of the workload. Maybe something that flashes up on the computer; that flashes up at me saying: ‘you've been working for this length of time, you know move now’ … .” (Participant 20, male, in their 40’s).*

*“I think you just forget yourself trying to beat the deadline! Probably if there was something that prompts, like setting an alarm on your phone or receiving a message on your phone to prompt you to move” (Participant 11, female, in their 40’s).*

### Opportunity

The participants identified some social opportunities that come from the TDF domain *social influences*, including restricting their colleagues from making tea for them to encourage them to get up more often to do it themselves, being part of a team to provide collective support and ensure a collective target is set, appointing someone like a fire marshal to remind people, or having walking and standing meetings.

*“Again I suppose it would have to come from another person to sort of tell me, that ‘you have got to remember that you need to stand’ I think someone like a fire marshal would get the job done (smiles)” (Participant 2, female, in their 40’s).*

*“If it was a corporate activity, I am more likely to engage with it. If you are on your own, you are less likely to do it. Being encouraged by other people would help a great deal” (Participant 23, female, in their 50’s).*

However, a popular opportunity amongst the participants was the need for an organisational culture that supports breaking up sitting to reassure employees that they will not be penalised if they stand up or leave their seat for a short while:

*“It's about the whole [organisation] being aware of true key messages, I think it's about promoting positive culture of movement. And that comes through communication, variety of communication strategies, it’s about communicating every opportunity about good practice about healthy movement … . and I guess it's about being given permission” (Participant 19, male, in their 30’s).*

*“Just knowing that my manager is okay with me getting up every half hour should be enough really. Apart from that, I’m okay but it’s a busy period right now so I have to be on my desk … . I get that, so if my manager is okay with me standing up, going back and forth for two to three minutes then coming back, then it’s fine” (Participant 3, female, in their 20’s).*

Creating the opportunity to influence the TDF domain *environmental context and resources* if cost was not a concern was highlighted by participants who suggested that a height-adjustable desk would be an important tool that could reduce their sitting in the workplace:

*“I think a raising desk is something that is worth exploring, but I understand that financially that is a huge investment for the [organisation] but there has been a lot of studies into that … . If money was not a problem, you can get raising desks, you can have it raised or seated and I will be happy to try that” (Participant 8, male, in their 30’s).*

### Motivation

Participants stated that the intervention should target both **reflective** and **automatic motivation** for behaviour change to take place. With regards to reflective motivation, around half of the participants reported that they felt in control of breaking up their sitting time, reflecting self-efficacy beliefs within the *beliefs about capabilities* TDF domain. For instance:

*“On a scale of ‘1’ to ‘10’, with ‘10’ being the most confident; I would say my confidence level [to sit less] is ‘8’” (Participant 16, male, in their 50’s).*

However, laziness and lack of will power was seen as a counter argument that may prevent them from doing so. In response, the participants highlighted they will need to change their mindset for a stronger commitment towards integrating movement and standing into their work life, which corresponds to the *intention* TDF domain.

*“The right mindset! That's what I need to be able to stand up and walk at regular intervals” (Participant 22, female, in their 40’s).*

Moreover, participants stated that they would respond to set goals if there was an expectation that they would be rewarded at the end, highlighting *goals* and *reinforcement* as important TDF domains.

*“Well, I'm motivated by having a pound every time I get up, or, or a chocolate every time I get up … It wouldn't necessarily have to be money, it could be a, as I say, a kind of build credits for some sort of treats … ” (Participant 25, female, in their 40’s).*

With respect to automatic motivation, the majority of the participants reported mixed perception about the effect of mood on their sitting time. Some participants said mood had no effect on their sitting time, while some thought it did. Either way, *emotion* appeared to be an important TDF domain that should be targeted.

*“My job determines my sitting behaviour, but my mood doesn’t – no!” (Participant 10, female, in their 40’s).*

*“It’s two ways: sometimes when I am happy I tend to be quite chatty, so I move more to talk to people, but when I’m low in mood I can sit all day at my desk or move more keeping to myself” (Participant 6, male, in their 20’s).*

Participants who perceived sitting time could be influenced by mood expressed that their optimism and motivation could be improved by having access to empirical evidence regarding the negative consequence of prolonged sitting.

*“Generally, people value research evidence, statistics, so in terms of increasing motivation and hope, informational literature on consequence of excessive sitting I guess will make a difference” (Participant 23, female, in their 50’s ).*

Participants also reported that they are likely to overcome the habit of sitting if there was competition among peers or if they were given incentives, underlining *reinforcement* as an important TDF domain.

*“You could develop some sort of challenge type thing. Erm, you know, people like games or competitions or even being given vouchers. People can find that quite motivating from that point of view” (Participant 6, male, in their 20’s).*

## Steps 5 and 6: identification of intervention functions and policy categories

Seven out of nine intervention functions described in the BCW guide [37] were identified as relevant based on the outcomes of the semi-structured interviews, mapped from COM-B shown in Table 1. These intervention functions are; *Education* (defined as increasing knowledge and understanding), *Training* (defined as imparting skills), *Persuasion* (defined as a way of using communication to stimulating positive or negative feeling or action), *Environmental restructuring* (defined as changing the physical or social context), *Enablement* (defined as increasing means and reducing barriers to increase capability), *Incentivisation* (defined as creating an expectation of reward), and *Modelling* (defined as providing an example for imitation).

**Table 1:** Combined link between COM-B model, TDF domains, intervention functions, policy categories and BCTs

COM-B Component	TDF	What needs to happen for the target behaviour to occur	Evidence to support the need for change (Quotes from the interviews)	Intervention Functions	Policy Categories	Behaviour Change Techniques (BCTs)
Psychological Capability	Knowledge	Have access to empirical evidence that supports breaking up sitting time	*“Personally, I think I base my decisions on evidence, case studies and ‘big shots’. Therefore, the more information we have about how it’s beneficial to people and exactly what has happened, the intervention provided and the exact result”* (Participant 24, female, in their 40's)	Education	Communication/Marketing, Guidelines	9.1 Credible source 5.1 Information about health consequences 5.3 Information about social and environmental consequences
		Have an awareness of the health consequence of excessive sitting	*“I think you need to keep spreading the message that sitting for long periods of time actually isn’t good for you”* (Participant 18, aged 59) *“I think, for me, possibly having a clearer understanding of the damage and negatives”* (Participant 6, male, in their 20's)	Education	Communication/Marketing, Guidelines	5.1 Information about health consequences
		Have an awareness of the benefit of breaking up sitting	*“I think it’s about … education, I think it’s about those key communication, and the positive, yes negative is important, but actually promoting the positive or focusing on the benefits rather than … ..”* (Participant 19, male, in their 30's)	Education	Communication/Marketing, Guidelines	5.1 Information about health consequences
		Have access to feedback about individual health behaviour	*“Erm, somebody from occupational health came out to see me, and pointed that I sit kind of wonky at my desk. I think if we have something or someone that tells us our progress, I’m sure everyone would be inclined to adjust”* (Participant 9, female in their 30's)	Education	Guidelines	2.2 Feedback on behaviour
		Know other strategies to break up sitting	*“Maybe by reducing use of emails a bit more; instead stand up and talk to people rather than email when they are just there”* (Participant 11, female in their 40's)	Education	Communication/Marketing	8.1 Behavioural practice/rehearsal 8.2 Behaviour substitution 8.3 Habit formation 8.4 Habit reversal
	Skills	Understand guidelines on sitting in the workplace	*“What do the experts say? Erm to be honest with you, I can’t say I have any particular knowledge or guidance to it. I don’t know what the recommendation is (smile) so I can’t answer that …*” (Participant 22, female in their 40's) *“I’m not aware of any advice that says ‘Don’t sit for longer than X amount”* (Participant 21, female in their 50's)	Training	Guidelines	4.1 Instruction on how to perform the behaviour
	Memory, Attention & Decision Processes	Improve ability to remember to take breaks from sitting	*“Yea I would do a chair that buzzes or causes electric shock; shaking chair that’s got a pressure pad on it so you know if it’s been sat on it for a long time”* (Participant 19, male in their 30's) *“I think technology can be used for pop-up on peoples’ computer every now and then, reminding them to get up and move or to get up and work once an hour or so”* (Participant 8, male, in their 30's)	Environmental restructuring Enablement	Environmental/Social planning	7.1 Prompts/cues 12.5 Adding objects to the environment 12.1 Restructuring the physical environment
	Behavioural Regulation	Identify and develop strategies to break existing habits and for self-monitoring of sitting	*If I’ve got something that I can look at and I think ‘oh I should be doing that’. The guilt factor always works … I would be willing to try if I know I’m being monitored* (Participant 10, female in their 40's) *“Getting drinks, getting water. For example, at the moment I do have a bottle on my desk but I’ve actually decided on getting a small cup to allow me stand up as many times as possible...” (*Participant 6, male, in their 20's)	Education Enablement	Communication/Marketing, Environmental/Social planning	2.3 Self-monitoring of behaviour 2.1 Monitoring of behaviour by others without feedback 2.2 Feedback on behaviour 1.2 Problem solving 1.4 Action planning 7.1 Prompts/cues 12.5 Adding objects to the environment
Physical Capability	Skills	Have physical strength to move more and sit less	*“Erm, if somebody was ill - I’m not personally, but - if somebody was, if they had a bad back or bad legs and it’s difficult for them to walk around on a regular basis, I think they would benefit a lot from getting help from physio and weight training”* (Participant 4, female, in their 50's)	Training Enablement	Environmental/Social planning	12.6 Body changes
Social Opportunity	Social influences	Have the enablement to make tea by oneself rather than by colleagues	*“Because they (colleagues) make my tea for me (laughs). We share the roles, we’ve got rota for making tea, so the four of us that drink tea take turns to get the drink. They are influencing my sitting time because they are making my drinks, so I’m not actually having to get up and do it myself”* (Participant 20, male, in their 40's)	Enablement	Environmental / social planning	6.3 Information about others’ approval 1.2 Problem solving 1.4 Action planning
		Consider creating a team for peer support and comparison	*“I would feel uncomfortable doing it on my own, so I just kind of carry on as I am, but I think if we were doing it as a whole, we would not feel alone and can compare what we are doing with our colleagues’”* (Participant 10, female, in their 40's)	Enablement	Environmental / social planning	6.2 Social comparison 12.2 Restructuring the social environment 3.1 Social support (unspecified)
		Identify a time keeper to get people moving	*“A possibility depends on whether I can get a Fire Marshall that would jump up and say ‘common people, let’s do stretches’. I think there are people in our office who are well placed to do that kind of thing”* (Participant 10, female, in their 40's)	Modelling Enablement	Environmental/Social planning	3.2 Social support (practical) 6.1 Demonstration of the behaviour
		Encourage having walking or standing meetings	*“Walking meeting would be nice. You know when you’re just walking around, having a meeting instead of sitting in a place”* (Participant 13, female, in their 20's)	Enablement	Environmental/Social planning	8.1 Behavioural practice/rehearsal 8.2 Behaviour substitution 8.3 Habit formation 8.4 Habit reversal 12.2 Restructuring the social environment
		Consider stretching or walking for 5 min every hour	“*Go for a walk every hour or do the stretching kind of every half an hour for five mins”* (Participant 9, female, in their 30's)	Enablement	Environmental/Social planning	1.1 Goal setting (behaviour) 1.4 Action planning
		Encourage senior management to participate in breaking up sitting to ensure support	*“I guess a manager would be appropriate person, so that you don’t feel you are doing something you should not do”* (Participant 24, female, in their 40's)	Enablement	Environmental/Social planning	3.1 Social support (unspecified) 12.2 Restructuring the social environment
		Organisational support for moving more and sitting less	*“I think there can be some sort of support from management or line managers to make sure that, you are not just sitting there continuously … Cultural change at higher level, maybe* via *a training section, leaflet or booklet that go around or one of those online courses that we normally do - like fire awareness training, health and safety training …*” (Participant 7, male, in their 30's)	Enablement	Environmental/social planning	3.1 Social support (unspecified) 12.2 Restructuring the social environment
Physical Opportunity	Environmental context and Resources	Provision of computer reminder system	*“I think technology can be used for pop-up on peoples’ computer every now and then, reminding them to get up and move or to get up and work once an hour or so”* (Participant 8, male, in their 30's)	Enablement	Environmental/Social planning	7.1 Prompts/cues 12.5 Adding objects to the environment
		Provide height-adjustable desks to ensure employees continue working while standing up	*“We probably do need our desks to be adjusted …*. *you know, at the right height. Well, I’m surprised this place doesn’t have them but I have worked in places where, hmm, where we have actually had height-adjustable desks. This place should have them, full stop”* (Participant 4, female, in their 50's)	Environmental restructuring	Environmental/Social planning	12.1 Restructuring the physical environment 12.5 Add object to the environment
		Move printers, water dispensers away from employees’ desks	*“Moving photocopiers and water dispenser further away … Same with toilet facilities. We’ve got to walk to them! Also, probably getting rid of all the rest of the printers, and we’ve only got one printer to use”* (Participant 20, male, in their 40's)	Environmental restructuring	Environmental/Social planning	12.1 Restructuring the physical environment
		Provide treadmill/ stand up chairs or buzzing chairs	*“Yeah I would do a chair that goes up and down or a chair that buzzes or causes electric shock; shaking chair that’s got a pressure pad on it so you know if it’s been sat on it for a long time”* (Participant 19, male, in their 30's) *“Mind you there are some brilliant chairs around, have you seen some of these new chairs, the stand-up ones, they are like rockers, and you’ve got to keep your stability and your muscles working …*. *‘cause your legs are permanently keeping you stable and those flexing which are equivalent of walking, but you’re not stood up”* (Participant 8, male, in their 30's)	Environmental restructuring	Environmental/Social planning	12.1 Restructuring the physical environment 12.5 Adding objects to the environment 7.1 Prompts/cues
		Access to a standing hot desk	*“Yeah, possibly a hot-desking idea might be a good one, switching from my desk to a higher one. Yeah good use for that!”* (Participant 13, female, in their 20's)	Environmental restructuring	Environmental/Social planning	12.1 Restructuring the physical environment 12.5 Adding objects to the environment
Reflective Motivation	Beliefs about Capabilities	Have a strong will and belief you can break up sitting	*“Somewhat confident, not massively … I hardly move until lunch break … I could break it up a little more and but not massively”* (Participant 8, male, in their 30's) *“I am not making any excuse, but it is difficult for me at the moment to see how I can incorporate exercise into my day …*” (Participant 18, female, in their 50's)	Education Persuasion	Communication/Marketing	15.1 Verbal persuasion about capability 15.2 Mental rehearsal of successful performance 1.4 Action planning
		Acknowledge the need for self-discipline	*“If you discipline yourself to do something you can do it, if you have willpower …*.” (Participant 3, female, in their 20's)	Education Persuasion	Communication/Marketing	8.3 Habit formation 4.2 Information about antecedents 8.1 Behavioural practice/rehearsal
	Goal	Have breaking up sitting goals with an expectation of reward	*“Well I’m motivated by having a pound every time I get up, or, or a chocolate every time I get up … It wouldn’t necessarily have to be money, it could be a kind of build credits for some sort of treat or, I don’t know, half an hour of you know”* (Participant 25, female, in their 40's) *“I think people could become quite motivated if you could develop some sort of challenge thing. Erm, you know, people like games or competitions, people can find motivation from that point of view”* (Participant 6, male, in their 20's)	Incentivisation	Communication/Marketing	1.1 Goal setting (behaviour) 1.2 Problem solving, 1.4 Action planning 10.1 Material incentive (behaviour) 10.2 Material reward (behaviour) 10.3 Non-specific reward 10.4 Social reward 10.5 Social incentive 10.6 Non-specific incentive 10.9 Self-reward
	Intention	Move from the state of contemplation to commitment to break up sitting	*“I just need to prioritize it really. It’s prioritization, you need that reminder”* (Participant 13, female, in their 20's)	Education Persuasion	Communication/Marketing	1.1 Goal setting 1.4 Action Planning
Automatic Motivation	Emotion	Discuss the risk involved in prolonged sitting to reduce the influence of mood	*“Because I’m low in mood I sit for a long time. Most times, when I leave I’m tired, lethargic, and drained. I think getting up more would just make me better by the end of the day”* (Participant 10, female, in their 40's)	Persuasion	Communication/Marketing	5.6 Information about emotional consequences 11.2 Reduce negative emotions 2.4 Self-monitoring of outcome(s) of behaviour 4.4 Behavioural experiments
	Reinforcement	Develop goals with incentives and reward to encourage employees to break up their sitting time	*“Maybe incentives, but I’m not sure what the incentive would be. Whether you do this and you get a bag of apples at the end of the month”* (Participant 10, female, in their 40's)	Incentivisation	Communication/Marketing	10.8 Incentive (outcome) 10.1 Material incentive (behaviour) 10.2 Material reward (behaviour) 10.3 Non-specific reward 10.6 Non-specific incentive

With respect to policy categories, only three out of the seven categories highlighted in the BCW guide [37] were identified. These included *Communication/marketing* (for instance, using verbal, electronic communication or flyers to create awareness of benefits of breaking up sitting and health consequences of prolonged sitting), *Guidelines* (examples of which include informing employees of sitting time guidelines), and *Environmental/social planning* (e.g, designing and controlling the logistics of height-adjustable desks within the office setting/office culture).

## Step 7: identification of behaviour change techniques

BCTs are considered as ‘active components’ when designing an intervention. In total, 39 out of the 93 BCTs in the BCT Taxonomy Version 1 [47] were identified from the interview data (Table 1). The list of BCTs identified include: ‘Instruction on how to perform the behaviour’, *‘*Credible source*’*, *‘*Information about health consequences*’*, *‘*Information about social and environmental consequences*’*, *‘*Feedback on behaviour*’*, *‘*Behavioural practice/rehearsal*’*, *‘*Behaviour substitution*’*, *‘*Habit formation*’*, *‘*Habit reversal*’*,*’* Prompts/cues*’*, *‘*Adding objects to the environment*’*, *‘*Restructuring the physical environment*’*, *‘*Self-monitoring of behaviour*’*, ‘Monitoring of behaviour by others without feedback’,*’* Problem solving*’*, *‘*Action planning*’*, *‘*Body changes*’*, *‘*Information about others’ approval*’*, *‘*Social comparison*’*, *‘*Restructuring the social environment*’*, *‘*Social support (unspecified)*’*, *‘*Social support (practical)*’*, *‘*Demonstration of the behaviour*’*, *‘*Goal setting*’*, *‘*Verbal persuasion about capability*’*, *‘*Mental rehearsal of successful performance*’*, *‘*Material incentive (behaviour)*’*, *‘*Material reward (behaviour)*’*, *‘*Non-specific reward*’*, *‘*Social reward*’*, Social incentive*’*, *‘*Non-specific incentive*’*, *‘*Self-reward*’*, *‘*Information about emotional consequences*’*, *‘*Reduce negative emotions*’*, *‘*Self-monitoring of outcome(s) of behaviour*’*, *‘*Behavioural experiments*’*, ‘Information about antecedents’ and *‘*Incentive (outcome)*’.*

Intervention designers will need to select BCTs that are most appropriate for the population and location where the intervention will be conducted. This can be achieved by considering the APEASE criteria or by first choosing BCTs that were most frequently used within relevant intervention functions before those that were less frequently used as described in the BCW guide [37].

## Step 8: mode of delivery

The appropriateness of mode of delivery depends on the target behaviour, target population and setting. Details on taxonomy of modes of delivery can be found in the BCW guide [37]. APEASE criteria should be used in selecting mode of delivery of choice. This could be either face-to-face or distance depending on setting. Where employees are spread over different offices and different locations, interventions could be delivered face-to-face, in clusters or individually. This can be achieved by giving out leaflets with detailed information about breaking up sitting, sitting guidelines for office workers and demonstrated using digital media.

## Discussion

The aim of this work was to use qualitative interviews with desk-based employees to highlight aspects of the BCW that can be used to develop a tailored intervention package that could be employed in breaking up and reducing workplace sitting. This research describes the systematic process used to model determinants of workplace sitting behaviour by qualitatively analysing sources of behaviour with the COM-B/TDF model, linking to subsequent intervention functions and policy within the BCW, and finally, identifying the appropriate behaviour change techniques to use when developing a tailored intervention to break up office workers’ sitting time. The majority of the participants in this study were not aware of any published recommendations for reducing sitting in the workplace [62]. However, participants expressed a keen interest in changing their sitting behaviour, suggesting that a workplace intervention targeted at sitting patterns would be acceptable.

The main reasons cited for prolonged sitting at work were the sedentary nature of the job, forgetfulness due to a heavy workload, an unsupportive physical workspace, and the organisational and social culture. These findings are consistent with previous studies that identified organisational cultural norms around “appropriate” workplace behaviour, environmental changes and workload pressures as barriers to breaking up workplace sedentary time [68–70]. The interview responses suggested that interventions should include education about sitting guidelines, health and emotional consequences of prolonged sitting and the benefits of reducing sitting time; prompts to serve as reminders to break up sitting; environmental modification, such as the provision of height-adjustable desks to alternate between sitting and standing without disrupting work; and changes to social and organisational support. Previous studies [71–73] have reported similar findings that breaks from prolonged sitting need to be seen as a “normal” activity in the workplace in order to prevent perceived criticism from colleagues. Organisational support would address this change. This could be an important strategy to prevent sedentary behaviour-induced diseases, due to a probable connection between social support, role-modelling, and social norms and the development of chronic diseases associated with prolonged sedentary behaviour [74].

In terms of the COM-B model, this study identified Psychological Capability, Social and Physical Opportunity as well as Reflective and Automatic Motivation as key targets for a behaviour change intervention for reducing and breaking up sitting time at work among office workers. In addition, the results from interviews with the participants suggested that *Knowledge, Skills, Reinforcement, Goals, Intentions, Environmental context and resources, Social influences, Behavioural regulation, Emotion*, and *Memory, attention and decision processes* were important TDF domains that need to be targeted in work-based sitting interventions. Consequently, seven intervention functions including *Education, Training, Modelling, Persuasion, Enablement, Environmental restructuring* and *Incentivisation* were identified as relevant for a sedentary workplace intervention. These results are in alignment with the SMArT study by Munir et al. [53] in which the BCW was also used to design a workplace sitting reduction intervention in hospital office workers. They identified the TDF domains of *Knowledge, Social identity, Intentions, Beliefs about capabilities, and Self-regulation of behaviour*, and consequently the key intervention functions of *Education, Enablement*, and *Training*. However, it should be noted that the present study identified a broader range of intervention functions due to the fact that the SMArT study applied the APEASE criteria to select the most relevant intervention function for the target population.

This study proposes 39 potential behaviour change techniques identified from this process. Several strategies that can be used to implement these behaviour change techniques include targeting cognitive memory by providing prompts and cues, offering rewards for successfully completing their target behaviour, providing information about breaking up sitting time and the consequences of prolonged sitting, providing access to height-adjustable desks, or reassuring employees of management support (see Table 2). Modifying the work environment through the introduction of active workstations has been found to effectively reduce sedentary behaviour in the workplace [75] without detrimental effects on work performance [76].

**Table 2:** Generalised recommendations for interventions based on interview with office workers

BCT code	Behaviour Change Techniques	Recommendations
3.1	Social support (unspecified)	Participants need to be assured that they have the support of their management and colleagues and that they will not be judged or punished for standing or leaving their desk to perform physical activity. This should increase their confidence to embrace the idea of taking breaks from sitting while at work.
7.1	Prompts/cues	On-screen computer prompts could be provided to serve as a reminder to take breaks from sitting.
1.1	Goal setting (behaviour)	Set a goal for participants to reduce prolonged sitting.
5.1	Information about health consequences	Provide information about the health consequences of prolonged sitting.
12.1	Restructuring the physical environment	To make breaking up sitting easier for the participants without necessarily leaving their desk, active workstations, such as height-adjustable desks should be provided to counteract employees’ and employers’ concern of losing productive time while standing up.
12.5	Adding an object to the environment	
6.1	Demonstration of the behaviour	Give detailed explanations on how to break up sitting time and demonstrate how to use equipment that is being provided, such as a height-adjustable desk or prompts.
4.1	Instruction on how to perform the behaviour	
4.2	Information about antecedents	Advise to keep a record of sitting and of events taking place before sitting.
3.2	Social support (practical)	Appoint someone to support office workers to reduce their sitting and demonstrate different forms of activities that could be done in the workplace.
8.1	Behavioural practice/rehearsal	Encourage office workers to replace sitting with walking or standing meetings and consider having face-to-face meetings instead of communicating by emails or intercoms.
8.2	Behavioural substitution	
8.3	Habit formation	
8.4	Habit reversal	
2.2	Feedback on behaviour	Feedback on sitting behaviour and progress should be provided to participants during the intervention to increase their motivation. This would enable them to review their action plans and goals.
12.2	Restructuring the social environment	Organise into clusters in such a way that participants are not isolated when given interventions to break up sitting. The set-up should be arranged such that they see other colleagues to promote support.
6.2	Social comparison	Ensure participants in the same office or cluster can take cues from their colleagues who may be taking regular breaks from sitting and compared changes in sitting time. Create a league table to share sitting data.
6.3	Information about others’ approval	Provide information about what others think of taking breaks from sitting. For instance, what they think about getting up by themselves to make a cup of tea instead of asking fellow colleagues to do this for them.
1.2	Problem solving	Participants should be encouraged to identify personal barriers to breaking up sitting and develop an action plan to overcome these barriers. For instance, getting up regularly for a drink or tea with a small cup instead of being served by colleagues or getting incentives or rewards for achieving goals.
1.4	Action planning	
10.1	Material incentive (behaviour)	Encourage participants to reward themselves in the future if they have been able to achieve to their goals. Also inform participants that they will be recognised and verbally congratulate them for achieving their daily sitting goals. Promise to reward participants with vouchers if they reduce their sitting time.
10.2	Material reward (behaviour)	
10.3	Non-specific reward	
10.4	Social reward	
10.5	Social incentive	
10.8	Non-specific incentive	
10.9	Incentive (outcome) Self-reward	
12.6	Body changes	Arrange physiotherapy or massage sessions for participants who have aching back or other parts of their body that is preventing them from reducing their sitting.
2.3	Self-monitoring of behaviour	Encourage participants to take notes of their daily postures at work or give a monitoring device that allows participants to track their sitting behaviour.
2.1	Monitoring of behaviour by others without feedback	Observe and record participants’ sitting behaviour without their knowledge.
9.1	Credible source	Present verbal, visual or written information about the consequences of prolonged sitting and benefits of breaking up sitting from researchers, government organisations or international bodies.
5.3	Information about social and environmental consequences	Provide information about how breaking up prolonged sitting has benefited office workers and other sets of people and the type of intervention provided.
5.6	Information about emotional	Inform the participants that excessive sitting can causes tiredness and lethargy whilst breaking up sitting may re-energise and increases concentration.
11.2	Reduce negative emotions	
2.4	Self-monitoring of outcome(s) of behaviour	Advise the participants to rate their wellbeing, weight and general health regularly (daily, weekly, every 2 weeks etc) to see the outcomes of reducing sitting time.
4.4	Behavioural experiments	The participants can experiment with taking breaks from sitting to see how it impacts their mood, energy, etc.
15.1	Verbal persuasion about capability	Boost employees’ morale by assuring them that they are capable of breaking up their sitting and that they should not give room for any self-doubts.
15.2	Mental rehearsal of successful performance	Advise employees to imagine taking breaks from sitting at work.

Consistent with the findings of this present study, Gardner, Smith [28] in their systematic review sub analysis of workplace interventions found 6 BCTs that frequently appeared in effective interventions to reduce sedentary behaviour: Review behavioural goals, Self-monitoring (behaviour), Instruction on how to perform behaviour, Information on health consequences, Behaviour substitution, and Adding objects to the environment. All but one of these BCTs, Review behavioural goals, was also found in the present study. The BCTs identified in this present study were identified from the qualitative data and it may be that Review behavioural goals was a BCT identified by interventionists from psychological theory sources. Therefore, when tailoring future interventions, researchers should consider including theoretically derived BCTs as well as those generated from the target population.

### Strengths of the study

This paper presents novel qualitative data following a detailed systematic process consistent with recommendations of the Medical Research Council, which requires every complex intervention development to undergo three different phases including theory, modelling and the experimental phase [29–31]. This present study on modelling, and statements from interviews have been theoretically-evaluated using COM-B/TDF as described in the BCW guide [37]. The barriers to breaking up and reducing sitting time identified in this current study and previous studies [68–70] are factors that operate at personal, social and environmental levels, which support a socio-ecological model of sedentary behaviour. This underlines the need for interventions to be targeted at multiple levels of influence on behaviour instead of targeting only individual, environmental or organisational factors. This current study goes beyond the socio-ecological model which only describes the levels at which to implement behaviour change strategies. Rather, this study identifies specific BCTs as ‘active ingredients’ which intervention designers can choose from and implement at relevant functional and policy levels in future workplace sedentary behaviour reduction interventions.

### Limitations of the study

Worthy to note is the fact that people with a history of musculoskeletal problems were excluded from this study, which could mean that the findings are not relevant to those with such conditions. This limited an analysis of Physical Capability from COM-B, which is not present in the results. Second, the subjectivity of the analysis must be acknowledged, as with many qualitative studies in addition to concerns over external validity due to a relatively small sample size. However, it is believed that the recruitment of participants from two different office settings as well as the rigour applied to the study process and data analysis, suggests that the findings might be transferable to other sedentary office settings. Differences in participants’ demographics could introduce bias, however, a probable population heterogeneity effect would have been minimised by purposively targeting participants who were all desk-based office workers with self-reported high level sitting of at least 5.5 h per workday. Furthermore, despite the clear framework and direction available on the use of the BCW, the process itself was lengthy and time-consuming, particularly the coding of BCTs from the qualitative interviews and the elements of COM-B and the TDF derived from the data. Whilst efficiency of use appears to be a limitation presently, developments in machine learning will soon mean the tool is more accessible [77].

## Conclusions

This study has identified possible components of a workplace intervention to break up and reduce sitting behaviour in the workplace based on the needs of office workers. This study emphasises the need for interventions to be targeted at multiple levels of influence on behaviour. Consequently, 39 BCTs have been identified and can be used as active ingredients in preparation for targeting the key determinants (Psychological Capability, Physical and Social Opportunity and Reflective and Automatic Motivation) of sitting behaviour in the workplace. Sedentary behaviour intervention designers should apply the APEASE criteria to determine the most appropriate intervention functions, policy categories and BCTs to use, drawing on the evidence presented here that identifies what needs to change. Future research can use the insight and modelling from this paper to test the effectiveness of an intervention based on the findings presented here, during an experimental phase as suggested by the Medical Research Council. This next phase could then provide an empirical basis for sitting behaviour policy implementation in the workplace.

## Data Availability

The datasets during and/or analysed during the current study available from the corresponding author on reasonable request.

## Abbreviations

APEASE: Affordability, Practicability, Effectiveness and Cost-effectiveness, Acceptability, Side effects/safety and Equity
BCTs: Behaviour change techniques
BCW: Behaviour Change Wheel
COM-B: Capability, Opportunity, Motivation – Behaviour
MVPA: Moderate-to-vigorous physical activity
TDF: Theoretical Domains Framework
